# The CAD risk locus 9p21 increases the risk of vascular calcification in an iPSC-derived VSMC model

**DOI:** 10.1186/s13287-021-02229-5

**Published:** 2021-03-06

**Authors:** Anja Trillhaase, Beatrice Schmidt, Marlon Märtens, Undine Haferkamp, Jeanette Erdmann, Zouhair Aherrahrou

**Affiliations:** 1 Institute for Cardiogenetics, University of Luebeck, Ratzeburger Allee 160, 23562 Luebeck, Germany; 2 DZHK (German Centre for Cardiovascular Research), Partner Site Hamburg/Kiel/Luebeck, Luebeck, Germany; 3 University Heart Centre Luebeck, 23562 Luebeck, Germany; 4 Fraunhofer Institute for Molecular Biology and Applied Ecology (IME), 22525 Hamburg, Germany

**Keywords:** Coronary artery disease, Vascular calcification, 9p21, iPSCs, VSMCs

## Abstract

**Background:**

Coronary artery disease (CAD) is the leading cause of death worldwide. Chromosome locus 9p21 was the first to be associated with increased risk of CAD and coronary artery calcification (CAC). Vascular calcification increases the risk for CAD. Vascular smooth muscle cells (VSMCs) are one of the major cell types involved in the development of vascular calcification.

**Methods:**

So far, mainly animal models or primary SMCs have been used to model human vascular calcification. In this study, a human in vitro assay using iPSC-derived VSMCs was developed to examine vascular calcification. Human iPSCs were derived from a healthy non-risk (NR) and risk (R) donor carrying SNPs in the 9p21 locus. Additionally, 9p21 locus knockouts of each donor iPSC line (NR and R) were used. Following differentiation, the iPSC-derived VSMCs were characterized based on cell type, proliferation, and migration rate, along with calcium phosphate (CaP) deposits. CaP deposits were confirmed using Calcein and Alizarin Red S staining and then quantified.

**Results:**

The data demonstrated significantly more proliferation, migration, and CaP deposition in VSMCs derived from the R and both KO iPSC lines than in those derived from the NR line. Molecular analyses confirmed upregulation of calcification markers. These results are consistent with recent data demonstrating increased calcification when the 9p21 murine ortholog is knocked-out.

**Conclusion:**

Therefore, in conclusion, genetic variation or deletion of the CAD risk locus leads to an increased risk of vascular calcification. This in vitro human iPSC model of calcification could be used to develop new drug screening strategies to combat CAC.

**Supplementary Information:**

The online version contains supplementary material available at 10.1186/s13287-021-02229-5.

## Background

Coronary artery disease (CAD) is the leading cause of death worldwide [1]. Atherosclerosis, one of the major complications of CAD, is caused by a buildup of plaque in the coronary arteries. Common risk factors for CAD include hyperlipidemia, hypertension, diabetes, obesity, and smoking, as well as increasing age [2, 3]. Additionally, it is estimated that CAD has a heritability factor of up to 50% [4, 5]. Genome-wide association studies (GWAS) have identified 163 genetic loci that are significantly associated with CAD [6]. Among these, the 9p21 locus was one of the first identified [7] and has been replicated by additional studies [8–10]. Furthermore, five CAD risk genes (9p21, *ADAMTS7*, *PHACTR1*, *MRAS*, and *COL4a1/COL4a2*) are associated with coronary artery calcification (CAC) [11].

Vascular calcification is described as deposition of calcium phosphate (CaP) minerals, usually as hydroxyapatite or inorganic phosphate (Pi), in cardiovascular tissues [12]. Vascular calcification is associated with a three- to four-fold increase in the risk of cardiovascular mortality [13], making it an important disease marker. One of the major cell types involved in the vascular calcification process are vascular smooth muscle cells (VSMCs). During calcification, VSMCs lose expression of SMC-specific markers and start expressing osteogenic genes [13–21], which results in the deposition of bone-like minerals such as CaP in the extracellular matrix (ECM) [13–15, 21].

Primary VSMCs are challenging to use because they lose their phenotypic properties when cultured in vitro, making it difficult to conduct long-term in vitro experiments with these cells. Additionally, they require characterization before they can be transplanted in vivo [22]. Therefore, induced-pluripotent stem cells (iPSCs) are a promising alternative to study vascular disease progression without the need of repetitive surgery to harvest primary VSMCs.

Human iPSCs can be generated from a variety of adult somatic cell types [23], and they provide an almost unlimited source of regenerative cells [23]. Human iPSCs can be differentiated into most cell types present in the adult human body [24], including VSMCs. However, there are only a few published methods for differentiating VSMCs from iPSCs. More recently, more complex protocols for generating human iPSC-derived VSMCs have been published that allow lineage-specific [25] or phenotype-specific [26] differentiation of VSMCs. Nevertheless, to date, there is no published protocol for differentiating iPSCs into calcifying vascular cells.

## Materials/methods

### Cell culture

Human iPSCs were reprogrammed from peripheral blood mononuclear cells (PBMCs) obtained from a healthy non-risk (HE463_7; NR) and a risk (C512, R) donor as previously described [27]; the latter carried SNPs in the 9p21 CAD risk interval. The authors of this previous study kindly provided the R, NR, and KO iPSC lines for the present study [27]. Human iPSCs of the four genotypes (R WT, NR WT, R KO, NR KO) were cultivated in mTeSR1 (StemCell Technologies) medium on Geltrex-coated dishes (Geltrex hESC qualified, ready-to-use, GFR, Thermo Fisher). Medium was exchanged every day. Additionally, a healthy control cell line, UCSD018i-3-6 (WiCell; referred to as 18i-3-6), was used as internal control and cultured as mentioned above.

Differentiation of iPSC into lateral-mesoderm (LM)-derived VSMCs was performed as described previously, with slight modifications [25]. Briefly, iPSCs were cultured in mTeSR1 and switched to maintenance medium (CDM-BSA) supplemented with 12 ng/mL FGF2 and 0.1 ng/mL Activin A for 1 d. Next, the cells were replated (d0) at a lower cell density than used in the original protocol [25] because a 1:2 to 1:3 splitting ratio was found to improve cell viability during mesoderm differentiation. Pluripotent cells were differentiated into early mesoderm properties within 1.5 d by supplementation with 20 ng/mL FGF2-IS, 10 μM LY294002, and 10 ng/mL BMP4, as reported by Cheung and colleagues. Between d1.5 and d5, the cells were differentiated into LM cells by adding 20 ng/mL FGF2-IS and 0.5 ng/mL BMP4. On d5, the cells were replated according to the published protocol. However, cell viability was improved by completely replenishing the medium every second day instead of replacing only 50%. From d5 until d18, the medium was supplemented with 10 ng/mL PDGF-BB and 2 ng/mL TGFβ1 [25]. For all cell lines, except the NR WT line, cells had to be passaged at a 1:2 to 1:3 ratio between d8 and d14. In the NR WT line, we did not observe the necessity to passage during differentiation. The resulting VSMCs were cultured on 0.1% gelatin-coated flasks in SMC maintenance medium (450 mL DMEM with high Glutamax + 50 mL FCS Gold Plus + 5 mL Pen/Strep; referred to as SMC medium). Medium was exchanged twice a week.

### Calcification

For calcification, VSMCs derived from the R WT, R KO, and NR KO iPSC lines were replated onto gelatin-coated 24-well plates at a density of 25,000–35,000 cells/well, or 50,000–55,000 cells/well for NR WT-derived VSMCs. At approximately 85–90% confluency, the calcification protocol was initiated. SMC maintenance medium was supplemented with the calcifying agents, as previously reported [28]. Medium was exchanged every 2–3 days. After 7 days, the calcification protocol was terminated, and the cells were used for immunostaining and RNA extraction.

### Immunofluorescence (IF) staining

Detection and localization of the target proteins within cultured iPSCs or iPSC-derived VSMCs was performed by immunofluorescence staining. For IF analysis, cells were grown on Nunc™ Lab-Tek™ II Chamber Slides™ (Thermo Scientific, #154534) at a seeding density of 0.5 × 10^4^ to 2 × 10^4^ cells per cm^2^. Cells were washed with PBS and then fixed for 30 min (mins) with chilled (− 20 °C) methanol to acetone (1:1). A Dako Pen (Dako, #S2002) was used to outline the wells to prevent antibody solutions from merging. For antibody staining, cells were permeabilized in PBS containing 0.1% Triton-X100 (Sigma Aldrich, #T-8787) and 1% BSA, then blocked with PBS containing 3.5% BSA. Next, 100 μL of primary antibody diluted in PBS (1:300–1:500), or PBS alone (negative control), was applied to each well and incubated overnight at 4 °C (Table 1).

**Table 1:** Antibodies used for IF

Antibody	Host species	Dilution	Manufacturer	Cat. number
hCNN1	Mouse	1:500	Sigma	C2687
hKi67 (marker of proliferation Ki67)	Mouse	1:300	BD Pharmingen	556003
hNANOG	Rabbit	1:300	Cell Signaling	4903
hTAGLN	Rabbit	1:500	Abcam	ab14106
Anti-rabbit IgG	Donkey	1:300	Invitrogen	A21206
Anti-mouse IgG	Goat	1:500	Invitrogen	A11029
Anti-mouse IgG	Donkey	1:500	Invitrogen	A10037

Slides were washed three times with PBS (each for 5 min) and then incubated for 1 h at room temperature with a secondary antibody (1:300–1:500) diluted in PBS (Table 1). Secondary antibodies were conjugated to a fluorescent dye (Alexa Fluor®) for target protein detection.

Subsequently, cells were washed three times with PBS, each for 5 min. To counterstain the nucleus of the cells, the blue-fluorescent stain 4′,6-diamidino-2-phenylindole (DAPI) was used, which binds to double-stranded DNA. Slides were incubated in the dark for 15 min with 0.1 μg/mL DAPI solution and washed with deionized water. The slides were covered using Dako Fluorescence mounting medium (Dako, #S3023) and glass cover slips (Menzel, #9161060). Detection of IF staining was performed using a BZ-9000 BioRevo™ fluorescence microscope (Keyence, Neu-Isenburg, Germany).

### Proliferation assay

Cells were stained with the Ki67 antibody according to the IF protocol. Nuclei were counterstained with DAPI. DAPI+ and Ki67+ cells were counted and the proportion of Ki67+ cells was compared with the overall number of cells (DAPI+). Results are presented as the percentage of Ki67+ cells in at least three independent biological replicates.

### Migration assay

For the scratch assay, VSMCs were seeded into a 12-well plate and cultured until confluent. Using a 100 μL tip, the cell layer in each well was scratched vertically. A single phase-contrast image was taken at previously specified positions in each well using an Olympus IX70 inverted microscope and CellSens Software. After 24 h, new images were taken at the same positions. Using the CellSens Software, the Confluency Checker tool was used to calculate the confluency at 0 h and 24 h. All time points included in the experiment were processed and confluency was analyzed. The difference between the confluency of 0 h and 24 h was set equal to the migration rate in %. Three independent biological replicates were analyzed.

### ARS and Calcein staining

Calcium phosphate (CaP) deposition was detected by Calcein (C30H26N2O13), a fluorescent chromophore that binds specifically to Ca^2+^ in cultured iPSC-VSMCs (Du et al., 2001). Cultured cells were washed once with PBS and fixed in chilled (4 °C) 4% paraformaldehyde (PFA) for 30 min. Cells were then incubated for 30 min in the dark with Calcein staining solution (Sigma, #C0875), washed three times with 50 mM TBS (pH 9) (each for 3 min), and then counterstained with DAPI for 10 min in the dark. Cells were covered with 400 μL of 50 mM TBS (pH 9), and Calcein fluorescence was detected using a BZ-9000 BioRevoTM fluorescence microscope (Keyence, Neu-Isenburg, Germany) immediately after staining. Alizarin Red S (ARS) staining was performed using the osteogenesis quantitation kit (Merck Millipore, # ECM815), following the manufacturers’ instructions. Stained cells were imaged using the BZ-9000 BioRevo™ fluorescence microscope (Keyence, Neu-Isenburg, Germany).

### Calcein quantification

Using a python script (Appendix), kindly provided by Dr. Tobias Reinberger from the Institute for Cardiogenetics, Luebeck, DAPI and Calcein intensity, and the pixel count, were estimated. The Calcein intensity was then normalized to DAPI (Intensity Calcein/DAPI pixel count). Untreated VSMCs were used as a reference.

### RNA isolation and quantitative polymerase chain reaction (qPCR)

mRNA was isolated from cells using the Qiagen RNEasy Mini Plus Kit (Qiagen, #74136), including DNase I treatment, following manufacturers’ instructions. RNA concentration and purity were determined using a BioPhotometer (Eppendorf, Hamburg, Germany), and samples were stored at − 80 °C until required. Synthesis of complementary DNA (cDNA) was performed via reverse transcription as follows: 10 μL of RNA, adjusted to a concentration of 100 ng/μL in DNase/RNase-free water (Gibco, 10977035), was incubated for 5 min at 68 °C. Next, 10 μL of reaction mix for reverse transcription, containing 4 μL 5× First Strand Buffer (Invitrogen, #28025021), 2 μL of 100 mM dithiothreitol (DTT) (Invitrogen, #28025021), and 1 μL each of 2′-deoxyribonucleosid-5′-triphosphates (dNTPs; 4 mM) (Promega, #U1330), Random Hexamer Primer-Mix (Roth, #HP28.1), RiboLock (40 U/μL) (Thermo Scientific, #EO0381), and M-MLV RT (200 U/μl) (Invitrogen, 28025021) were added to the RNA and incubated for 1 h at 37 °C, followed by enzyme inactivation at 95 °C for 5 min. Finally, the cDNA was stored at − 20 °C until further use. Quantitative gene expression analysis, known as PCR, was performed on cDNA using a 7900HT Fast Real-Time PCR System (Applied Biosystems, #4329001) and the corresponding SDS 2.2.2 software.

### Statistical analyses

All data points are represented as dot plots, unless otherwise stated. The median is represented by horizontal bars. Unpaired *t* tests using Welch’s correction were performed using GraphPad Prism 6.04. *P* < 0.05 was considered statistically significant. For all analyses, *P* values are indicated as significant (*P < 0.05, **P < 0.01, ***P < 0.001, ****P < 0.0001), or not significant (n.s.), unless stated otherwise. Statistic outliers were identified using the GraphPad outlier test and then excluded from the analyses.

## Results

### Differentiation of iPSCs into VSMCs and their characterization

Human iPSCs generated from a 9p21 risk (R) or a non-risk (NR) donor were differentiated into lateral mesoderm-derived VSMCs by slight modification of a previously published protocol (Cheung et al., 2014).

**Fig. 1:**
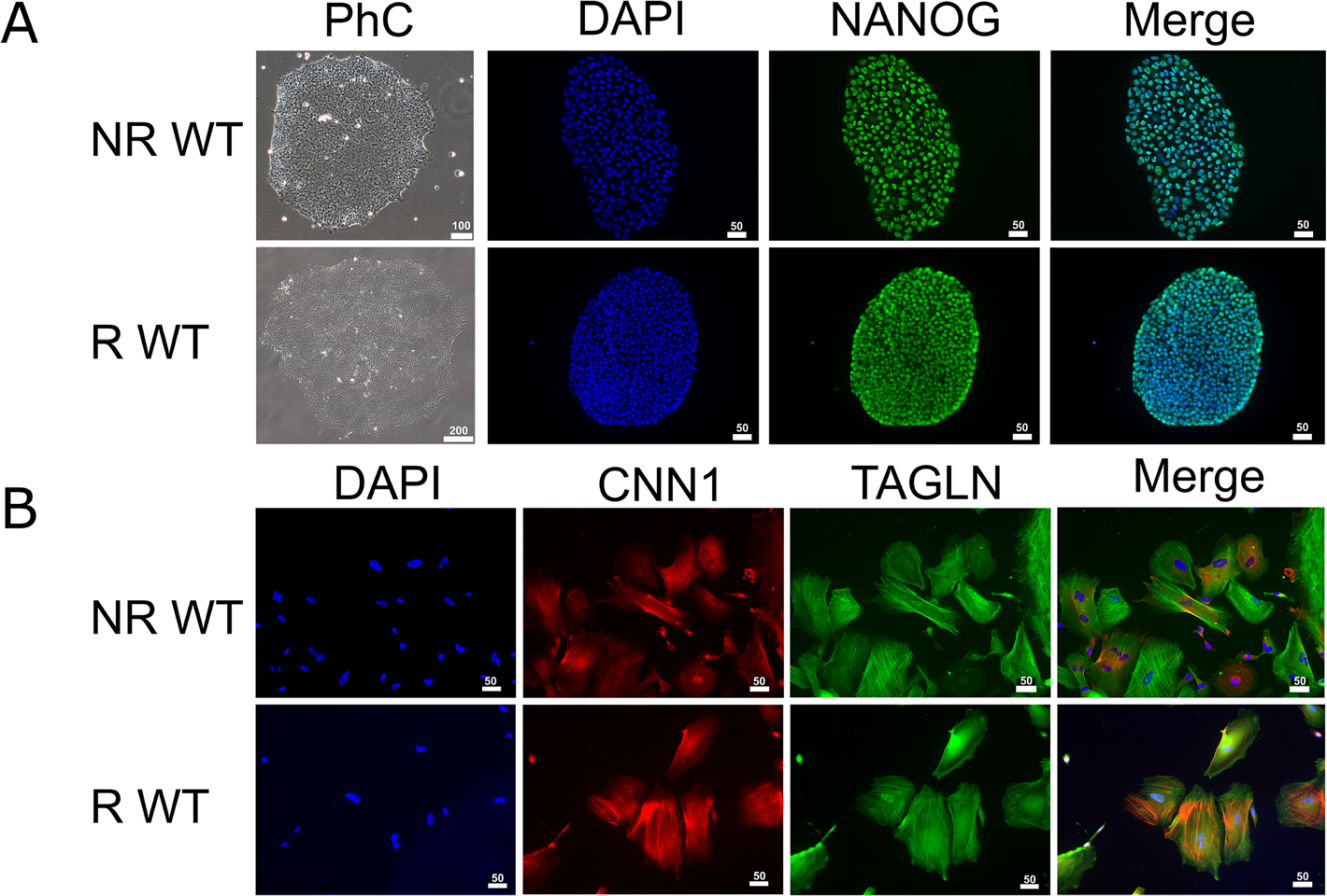
Characterization of WT iPSCs and derived VSMCs. **a** NR (top) and R (bottom) WT iPSCs show similar iPSC morphology, as seen in the phase contrast (PhC) images. Scale bars represent 100 μm. Staining with the pluripotency marker NANOG (green) confirmed nuclear expression, as seen in the overlaid image (merge). Nuclei were counterstained with DAPI (blue). Scale bars represent 50 μm. **b** NR (top) and R (bottom) WT iPSC-derived VSMCs show colocalization of CNN1 (red) and TAGLN (green) in the contractile apparatus of the cells. No differences were observed between R and NR WT VSMCs. Nuclei were counterstained with DAPI. Scale bars represent 50 μm

The undifferentiated iPSCs and their derived VSMCs were characterized based on morphology, where iPSCs show a typical colony growth with tightly packed cells, while VSMCs look star or stellate shaped. Further, RNA profile, protein expression, and localization were analyzed in both cell types. Phase contrast imaging revealed that the NR and R WT undifferentiated iPSCs had similar morphology (Fig. 1). Immunofluorescence (IF) staining with the pluripotency marker NANOG and nuclear co-stain DAPI showed clear nuclear protein localization (Fig. 1a). Overall, there were no differences in morphology or protein expression between the NR or R WT iPSCs. The 9p21 CAD risk locus was knocked-out from both R and NR donor iPSC lines (9p21 R and NR KO), as previously described [27] and then characterized using the same parameters. Both the 9p21 R and NR KO lines displayed typical morphologies, with identical nuclear localization of NANOG protein (Suppl. Figure 1).

The iPSC-derived VSMCs were characterized based on their morphology, protein expression, and protein localization. IF staining of CNN1 and TAGLN, two common SMC markers, revealed co-expression in the contractile fibers of iPSC-derived VSMCs. This was observed in both the NR- and R WT-derived VSMCs (Fig. 1b). No significant morphological or protein expression differences were observed between R and NR WT cells. Similarly, the 9p21 KO iPSC-derived VSMCs displayed normal morphology, and colocalization of CNN1 and TAGLN in the contractile fibers (Suppl. Figure 2). No differences were detected between the KOs from the R or NR donors.

**Fig. 2:**
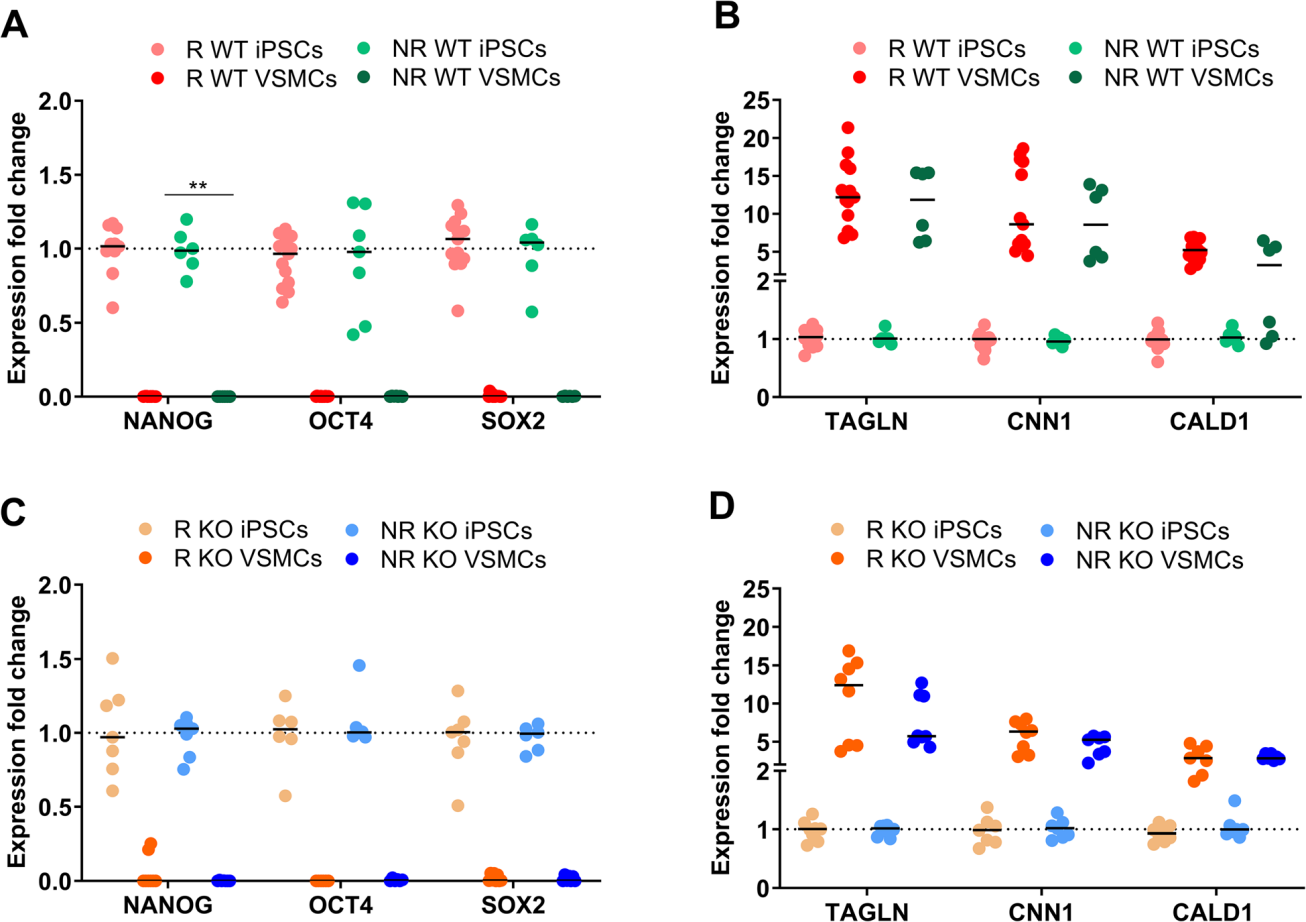
RNA expression analyses of pluripotency (**a**, **c**) and SMC-associated (**b**, **d**) genes. **a** Expression of pluripotency-associated markers *NANOG*, *OCT4*, and *SOX2* was lower in VSMCs derived from NR (green) and R WT (red) iPSCs than in undifferentiated iPSCs. **b** SMC-associated markers *TAGLN*, *CNN1*, and *CALD1* were upregulated in VSMCs derived from NR (green) and R WT (red) iPSCs compared with undifferentiated iPSCs. **c** NR KO (orange) and R KO (blue) lines showed significant downregulation of pluripotency genes *NANOG*, *OCT4*, and *SOX2*. **d** SMC-associated markers *TAGLN*, *CNN1*, and *CALD1* were upregulated in VSMCs derived from NR (green) and R WT (red) iPSCs compared with undifferentiated iPSCs

Additionally, expression of mRNAs encoding pluripotency- and SMC-specific genes was compared across all cell lines. The expression of NANOG, OCT4, and SOX2, were significantly decreased in NR and R WT derived VSMCs, compared to undifferentiated iPSCs (Fig. 2a). There were no significant differences between the R and NR cell lines. Expression of mRNAs encoding SMC-associated genes TAGLN, CNN1, and CALD1 was significantly higher in R- and NR WT-derived VSMCs than in undifferentiated iPSCs (Fig. 2b). NR KO- and R KO-derived VSMCs showed lower expression of pluripotency markers than iPSCs (Fig. 2c), while SMC markers were significantly upregulated in VSMCs compared with iPSCs (Fig. 2d). Overall, no significant differences in gene expression were observed between the genotypes. To validate the RNA results, we performed western blot analysis to detect expression of OCT4 and TAGLN proteins (data not shown).

### 9p21 affects iPSC-derived VSMC proliferation and migration

To further analyze iPSC-derived VSMCs, we examined their proliferation, migration, and calcification properties.

**Fig. 3:**
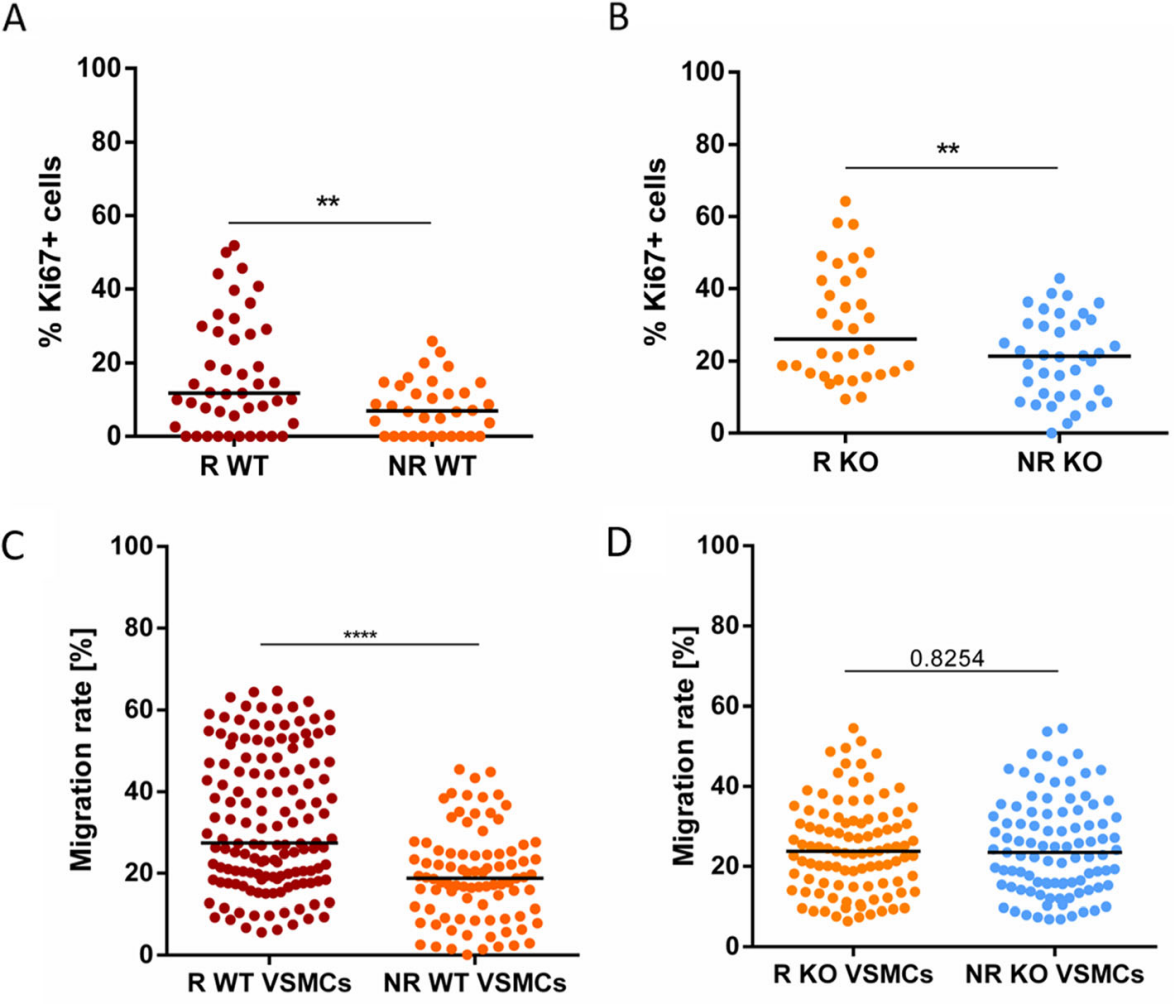
The 9p21 locus influences proliferation and migration of iPSC-derived VSMCs. **a** R WT (red) derived VSMCs showed significantly higher proliferation rates than those derived from NR WT (orange). **b** R KO (orange) VSMCs showed higher proliferation rates than those derived from NR KO (blue). **c** R WT (red) VSMCs showed significantly higher migration rates than those derived from NR WT (orange). **d** There were no differences in migration rate between R KO (orange) and NR KO (blue) VSMCs

First, to estimate the number of proliferative cells, Ki67 IF staining was performed. R WT VSMCs contained a higher amount of Ki67 positive (+) cells than NR WT VSMCs (Fig. 3a; Suppl. Figure 3). R KO VSMCs also contained more Ki67+ cells than NR KO cells (Fig. 3b; Suppl. Figure 4). Together, these results indicated increased proliferation of R donor iPSCs. Furthermore, the migratory capacity of iPSC-derived VSMCs was characterized in a scratch assay. R WT VSMCs showed a significantly higher migration rate than NR WT VSMCs (Fig. 3c; Suppl. Figure 5). However, there were no differences in migration rate between R and NR KO iPSC-derived VSMCs (Fig. 3d; Suppl. Figure 6).

### Testing and optimizing calcification protocols with iPSC-derived VSMCs

To identify the calcification protocol that was most suitable for our aim, we tested different calcification methods. First, the most common calcification method was tested, which involves supplementation with Pi or a combination of beta-glycerophosphate (β-GP) and L-ascorbic acid phosphate (L-AP). However, this did not result in positive calcification (data not shown). Next, the commercial StemXVivo® human osteogenic medium (R&D Systems, #CCM008/CCM007) was tested for 30 days. The initial trials in one cell line looked quite promising; however, when all cell lines were tested the calcification efficiency was only 20%. Next, the StemXVivo® medium was supplemented with TNFα, which achieved 25% calcification efficiency. Additionally, various combinations of H_2_O_2_ were combined with BMP2 and TNFα. However, H_2_O_2_ led to extensive apoptosis of iPSC-derived VSMCs, and addition of TNFα and BMP2 did not induce calcification. Next, two recently published protocols for in vitro calcification of human and mouse VSMCs were compared. The Alves and colleagues (2014) calcification cocktail comprised 0.1 mM L-AP, 10 mM β-GP, and 100 nM dexamethasone in either SMC or StemXVivo® medium containing 1.8 mM calcium chloride (CaCl_2_) and 20 mM HEPES [29]. When tested, this cocktail did not result in detectable calcification of iPSC-derived VSMCs. The final protocol comprised 4 mM CaCl_2_, 5 mM β-GP, 50 μg/ml L-AP, 1 μM insulin, and 0.1 μM dexamethasone in StemXVivo® medium for 7 d [28]. This protocol achieved an overall calcification efficiency of almost 40% (Suppl. Figure 7). Based on these tests, the Tziakas method (referred to as Tziakas cocktail) was chosen for further calcification experiments.

### Successful calcification of VSMCs derived from R and KO iPSCs

**Fig. 4:**
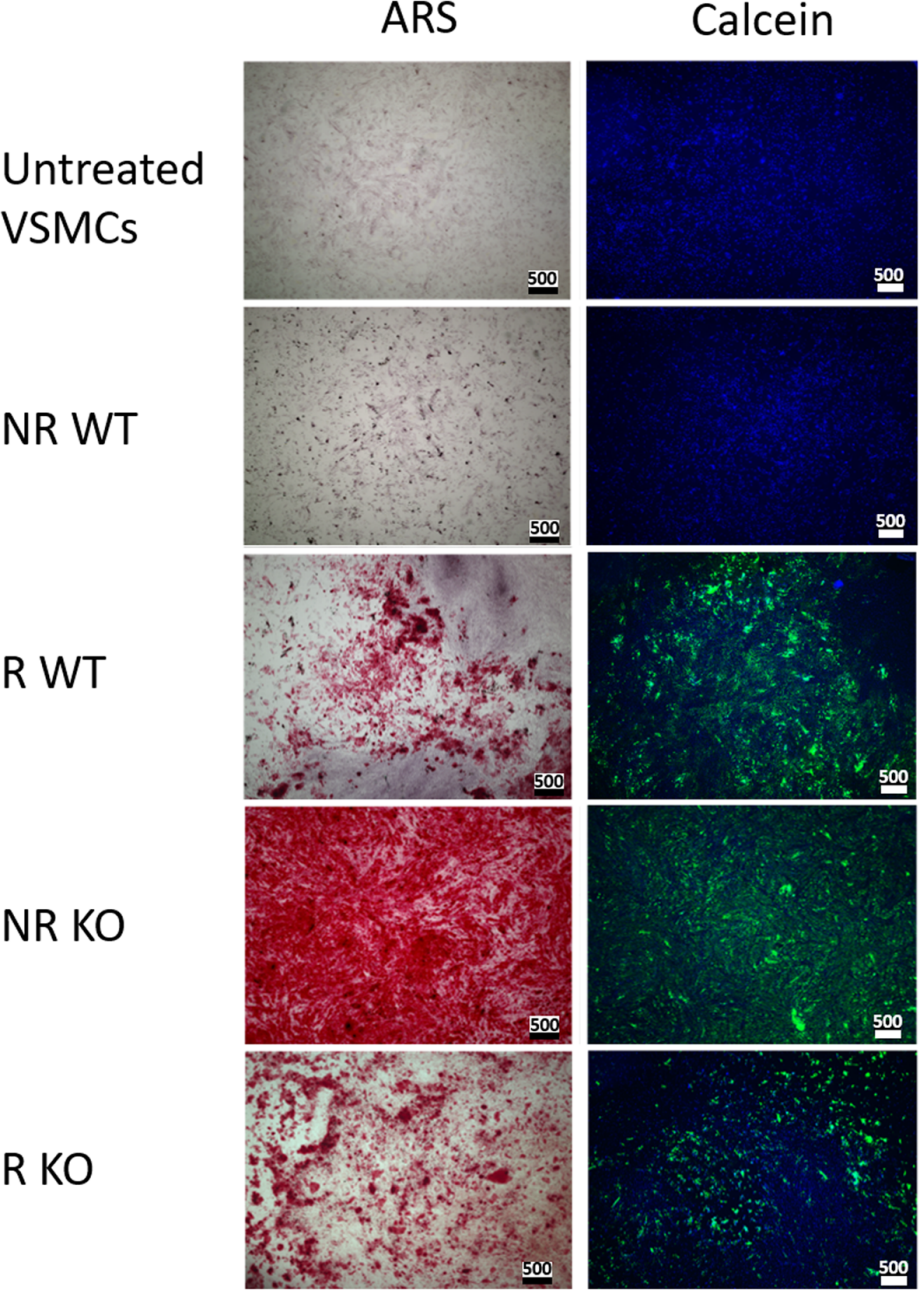
ARS and Calcein staining of calcifying iPSC-derived VSMCs. NR WT cells were negative for ARS (left) and Calcein (right) expression. R WT-, R KO-, and NR KO-derived VSMCs were positive for ARS (red, left) and Calcein (green, right), suggesting the presence of CaP deposits. Scale Bars represent 500 μm

Characterized iPSC-derived VSMCs were calcified by incubation for 7 days with the Tziakas cocktail as described previously [28]. To detect CaP deposits, the VSMCs were immunostained with ARS and Calcein (Fig. 4). NR WT iPSC-derived VSMCs were not positive for ARS or Calcein following treatment with the calcification cocktail. R WT-, NR KO-, and R KO-derived VSMCs contained CaP deposits, as determined by positive ARS staining or Calcein staining (Fig. 4).

The CaP deposits were quantified with a python script based on Calcein staining (Suppl. 1). R WT-derived VSMCs showed a significant increase in Ca after treatment with the calcifying cocktail, while NR WT-derived VSMCs showed no change in Ca content (Suppl. Figure 8A). VSMCs derived from either the R or NR KO lines showed a similarly significant increase in Ca levels (Suppl. Figure 8B).

**Fig. 5:**
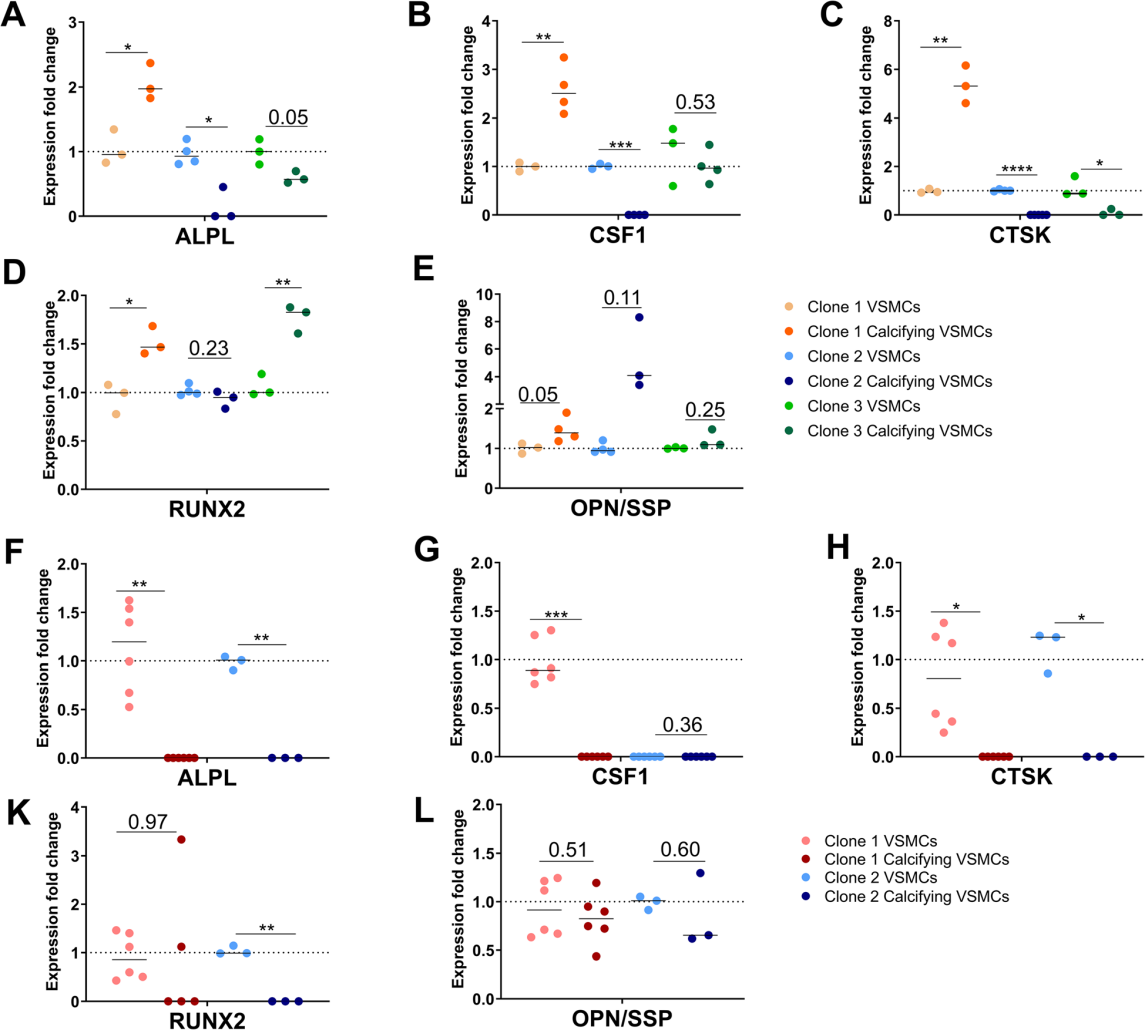
Calcification-associated markers are increased in R WT, but decreased in NR WT calcifying VSMCs. **a**–**e** R WT-derived calcifying VSMCs show increased expression of calcification markers *ALPL* (**a**), *CSF1* (**b**), and *CTSK* (**c**) (clone 1 (orange)) and decreased or unchanged expression in the other two clones (blue and green). *RUNX2* (**d**) was upregulated significantly in two out of three R WT clones. *OPN* (**e**) expression did not change significantly. **f–l** In NR WT-derived calcifying VSMCs, the markers *ALPL* (**f**), *CSF1* (**g**), and *CTSK* (**h**), and *RUNX2* (**k**) were downregulated in one or both clones. *OPN* (**l**) expression was unchanged in NR WT cells

Expression of calcification-associated markers *ALPL*, *CSF1*, *CTSK1*, *RUNX2*, and *OPN* was investigated in calcifying VSMCs across all iPSC lines. Some genes (*ALPL*, *CSF1,* or *CTSK1*) were upregulated in at least one clone of the R WT-derived VSMCs (Fig. 5). *RUNX2* was upregulated in two of three clones (Fig. 5d), while *OPN* expression was unchanged in all three clones of the R WT genotype (Fig. 5e).

The expression profile of the NR WT iPSC-derived VSMCs (Fig. 5f–l) was more distinct. Most of the markers were downregulated in calcifying VSMCs compared with untreated VSMCs. Only *OPN* was unchanged in the calcifying VSMCs derived from both NR WT clones (Fig. 5l).

**Fig. 6:**
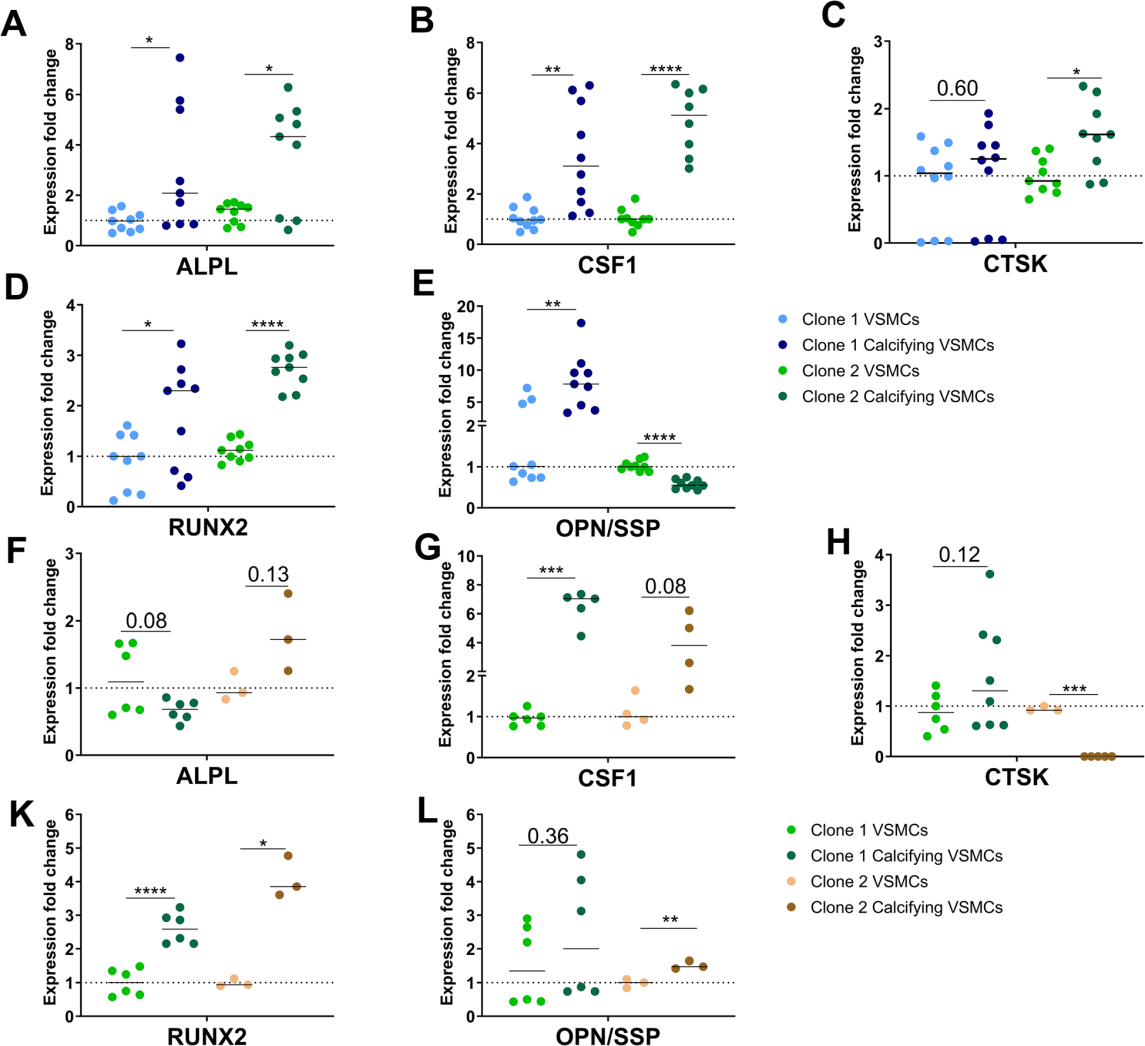
Upregulated expression of most calcification-associated markers in 9p21 KO calcifying VSMCs. **a–e** R KO-derived calcifying VSMCs showed upregulation expression of markers *ALPL* (**a**), *CSF1* (**b**), and *RUNX2* (**d**) in both clones. Expression of *CTSK* (**c**) and *OPN* (**e**) was either unchanged or downregulated in one clone of the R KO cells. **f–l** In the NR KO-derived calcifying VSMCs, the markers *CSF1* (**g**) and *RUNX2* (**k**) were upregulated in both clones. *ALPL* (**f**), *CTSK* (**h**) and *OPN* (**l**) were unchanged or downregulated in one clone of the NR KO cells

R KO-derived calcifying VSMCs showed significantly higher expression of *ALPL* (Fig. 6a), *CSF1* (Fig. 6b), and *RUNX2* than untreated VSMCs (Fig. 6d). Expression of *CTSK* (Fig. 6c) and *OPN* (Fig. 6e) was lower or similar in the R KO-derived VSMCs, whereas the genes *CSF1* (Fig. 6g) and *RUNX2* (Fig. 6 k) were upregulated in NR KO-derived calcifying VSMCs. The other markers were either downregulated or similarly expressed (Fig. 6f, h, and l).

Taken together, the gene expression patterns varied across the experiments and between genotypes. However, calcifying VSMCs derived from iPSCs with the 9p21 KO showed overall upregulation of most calcification markers. In conclusion, the NR WT-derived VSMCs did not calcify in response to the calcification stimulus. Meanwhile, calcifying VSMCs derived from the R WT, NR KO, and R KO iPSC lines showed signs of calcification and a partial increase in expression of calcification markers.

## Discussion

Cardiovascular disease is the number one killer among all other diseases worldwide. GWAS have associated the 9p21 locus with CAD, myocardial infarction (MI), and vascular calcification [7, 30, 31]. In this study, we obtained human iPSCs from a healthy donor and a CAD risk donor carrying genetic variants within the 9p21 locus. Furthermore, iPSC lines with the 9p21 locus knocked-out were generated [27]. To study vascular calcification, VSMCs were differentiated from the iPSC lines and then calcified. To our knowledge, this is the first establishment of an in vitro calcification model.

iPSCs were differentiated into lateral mesoderm-derived VSMCs by slight modification of a previously published protocol [25]. No differences were observed between undifferentiated or VSMCs derived from either WT or KO iPSC lines. Across all cell lines, the pluripotency markers *NANOG*, *OCT4*, and *SOX2*, as well as the SMC-specific markers *TAGLN*, *CNN1*, and *CALD1,* were expressed in iPSCs and VSMCs, respectively. Therefore, the 9p21 locus does not seem to affect iPSC or VSMC gene expression. The proliferation and migration rates of iPSC-derived VSMCs were analyzed because they are relevant parameters for atherosclerosis. R WT and R KO-derived VSMCs showed a higher proliferation rate than those derived from NR WT and KO lines. This result supports a previous report [27] showing that proliferation is higher in R WT-derived VSMCs, but that KO of the 9p21 locus restores the proliferation rate to NR WT levels [27]. It could be assumed that the observed differences were due to the use of different techniques to determine the proliferation rate. For example, Lo Sardo et al. (2018) used the number of passages at a given time as an indication of proliferation rate. In the present study, we used Ki67 staining to quantify the number of proliferative cells relative to the total cell count (obtained using the nuclear stain DAPI). Both methods have their technical subtleties, which may lead to contradictory results.

The VSMCs were analyzed further using a scratch assay to measure migration rate. The assay revealed an increased migration rate in R WT-derived VSMCs compared with those derived from NR WT iPSCs. There was no significant difference between R KO- and NR KO-derived VSMCs. Therefore, we were able to confirm that the 9p21 risk region influences VSMC behavior [27].

Moreover, the role of 9p21 in vascular calcification was assessed using a cellular-based assay. Previously, most in vitro calcification studies were conducted with either harvested mouse or human primary aortic SMCs or mesenchymal stem cells [28, 29]. However, the use of primary cells is limited by ethics and accessibility and further compounded by differences between animal models and the human system.

For human iPSC-derived VSMCs, we used a previously published protocol consisting of a cocktail of CaCl_2_, β-GP, L-AP, insulin, and dexamethasone to generate an in vitro model of calcification [28]. This protocol was 44% efficient with respect to calcification of iPSC-derived VSMCs from all cell lines (data not shown). NR WT-derived VSMCs showed no signs of calcification, neither by staining and quantification, nor by expression of associated markers *ALPL*, *CTSK*, *CSF1*, *RUNX2*, or *OPN*. R WT-, R KO-, and NR KO-derived VSMCs all contained Ca deposits, as determined by immunostaining and quantification analysis, with partial upregulation of calcification-associated markers. In conclusion, KO of the 9p21 CAD risk region leads to increased Ca deposition in VSMCs; therefore, patients carrying SNPs in this genomic locus are at greater risk for developing vascular calcification.

However, some variation was observed during the calcification experiments both between and within iPSC lines. The variable results may be due to the use of serum in the SMC maintenance and calcification media. Bovine serum contains anti-calcific agents such as fetuin-A, which decrease the calcification efficiency in vitro by up to 30% [32, 33]. Furthermore, it is not clear what influence different matrices have, or which exact SMC phenotype is induced by the differentiation protocol used. In future, it would be desirable to optimize the protocol to improve efficiency and reduce variability.

Nevertheless, the method presented in this study provides a good basis for investigating vascular calcification of VSMCs in vitro using patient-derived iPSCs. In future, it could be applied to investigate other CAD gene candidates to estimate the heritable risk of vascular calcification. The underlying mechanisms leading to calcification of iPSC-derived VSMCs remain unclear and need to be investigated in future studies. In particular, the balance between linear versus circular *ANRIL* RNA and related pathways, as recently reported by Holdt and coworkers, would be worth investigating [34].

## Conclusion

The 9p21 locus is associated with vascular calcification using GWAS. In this work, we aimed to go beyond GWAS and established a protocol to functionally study vascular calcification using iPSC-derived VSMC. We demonstrated increased proliferative and migratory ability of the 9p21 risk genotype in iPSC-derived VSMC, which are consistent with a recently published study. Furthermore, we demonstrated that the loss of or SNPs within the 9p21 CAD risk locus leads to increased vascular calcification in calcifying iPSC-derived VSMCs. Hence, patients carrying risk SNPs in the respective locus are of much higher risk of developing coronary calcification and thus coronary artery disease.

## Supplementary Information

**Additional file 1: Suppl. Figure 1.** The 9p21 locus does not influence iPSC morphology or protein localization. **Suppl. Figure 2.** The 9p21 locus does not influence VSMC morphology or protein localization. **Suppl. Figure 3.** Representative images of Ki67 staining in R vs NR WT VSMCs. **Suppl. Figure 4.** Representative images of Ki67 staining in R vs NR WT VSMCs. **Suppl. Figure 5.** Representative images of migration of R vs NR WT VSMCs. **Suppl. Figure 6.** Representative images of migration of R vs NR KO VSMCs. **Suppl. Figure 7.** Efficiency of Tziakas calcification cocktail over genotypes. **Suppl. 1.** Python Script for Calcein quantification (by Dr. Tobias Reinberger). **Suppl. Figure 8.** Calcein quantifications had increased Ca levels in R WT, and both KO genotypes.

## Data Availability

The data that support the findings of this study are available from the corresponding author upon reasonable request.

## Abbreviations

ARS: Alizarin Red Staining
CAC: Coronary artery calcification
CaCl_2_: Calcium chloride
CAD: Coronary artery disease
CaP: Calcium phosphate
cDNA: Complementary DNA
DTT: Dithiotreitol
ECM: Extracellular matrix
GWAS: Genome-wide association study
IF: Immunofluorescence
iPSCs: Induced pluripotent stem cells
KO: Knockout
L-AP: L-Ascorbic acid phosphate
LM: Lateral mesoderm
MI: Myocardial infarction
NR: Non-risk
PFA: Paraformaldehyde
qPCR: Quantitative polymerase chain reaction
R: Risk
SMCs: Smooth muscle cells
VSMCs: Vascular smooth muscle cells
WT: Wildtype
β-GP: Beta-glycerophosphate
