# Supplementary material for: The CAD risk locus 9p21 increases the risk of vascular calcification in an iPSC-derived VSMC model
Source: Stem Cell Res Ther. 2021 Mar 6;12:166. doi: 10.1186/s13287-021-02229-5 (PMC7936418; doi:10.1186/s13287-021-02229-5)
Supplement: Supplementary file 1 — Additional file 1: Suppl. Figure 1. The 9p21 locus does not influence iPSC morphology or protein localization. Suppl. Figure 2. The 9p21 locus does not influence VSMC morphology or protein localization. Suppl. Figure 3. Representative images of Ki67 staining in R vs NR WT VSMCs. Suppl. Figure 4. Representative images of Ki67 staining in R vs NR WT VSMCs. Suppl. Figure 5. Representative images of migration of R vs NR WT VSMCs. Suppl. Figure 6. Representative images of migration of R vs NR KO VSMCs. Suppl. Figure 7. Efficiency of Tziakas calcification cocktail over genotypes. Suppl. 1. Python Script for Calcein quantification (by Dr. Tobias Reinberger). Suppl. Figure 8. Calcein quantifications had increased Ca levels in R WT, and both KO genotypes. [file 13287_2021_2229_MOESM1_ESM.docx]

**Supplementary Material**

**The CAD risk locus 9p21 increases the risk of vascular calcification in an iPSC-derived VSMC model**

Trillhaase, Anja^1^; Schmidt, Beatrice^1^; Märtens, Marlon^1^; Haferkamp, Undine^2^;

Erdmann, Jeanette^1(*)^; Aherrahrou, Zouhair^1(*)^

(*) denotes equal contribution

^1^ Institute for Cardiogenetics, University of Luebeck, Germany; DZHK (German Centre for Cardiovascular Research), Partner Site Hamburg/Kiel/Luebeck, Germany; University Heart Centre Luebeck, 23562 Luebeck, Germany

^2^ Fraunhofer Institute for Molecular Biology and Applied Ecology (IME), 22525 Hamburg, Germany

Suppl. Fig. 1: The 9p21 locus does not influence iPSC morphology or protein localization.

Suppl. Fig. 2: The 9p21 locus does not influence VSMC morphology or protein localization.

Suppl. Fig. 3: Representative images of Ki67 staining in R vs NR WT VSMCs.

Suppl. Fig. 4: Representative images of Ki67 staining in R vs NR WT VSMCs.

Suppl. Fig. 5: Representative images of migration of R vs NR WT VSMCs.

Suppl. Fig. 6: Representative images of migration of R vs NR KO VSMCs.

Suppl. Fig. 7: Efficiency of Tziakas calcification cocktail over genotypes.

Suppl. 1: Python Script for Calcein quantification (by Dr. Tobias Reinberger)

Suppl. Fig. 8: Calcein quantifications had increased Ca levels in R WT, and both KO genotypes.

Suppl. Fig. 1


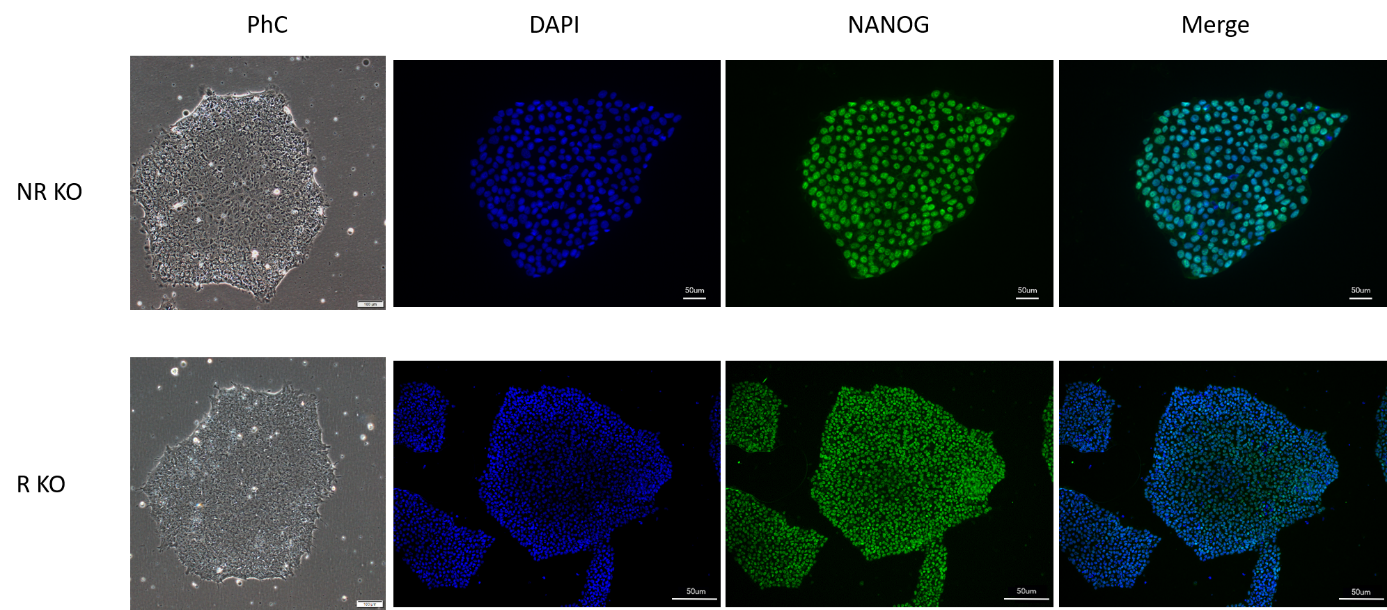


**Suppl. Fig. 1: The 9p21 locus does not influence iPSC morphology or protein localization.**

NR (Top) and R (bottom) KO iPSCs show typical morphology. IF staining of NANOG shows localization in the nucleus. There were no differences between R and NR KO-derived iPSCs. Nuclei were counterstained with DAPI. Scale Bars = 100 µm in the PhC image, and 50 µm in the IF images.

Suppl. Fig. 2


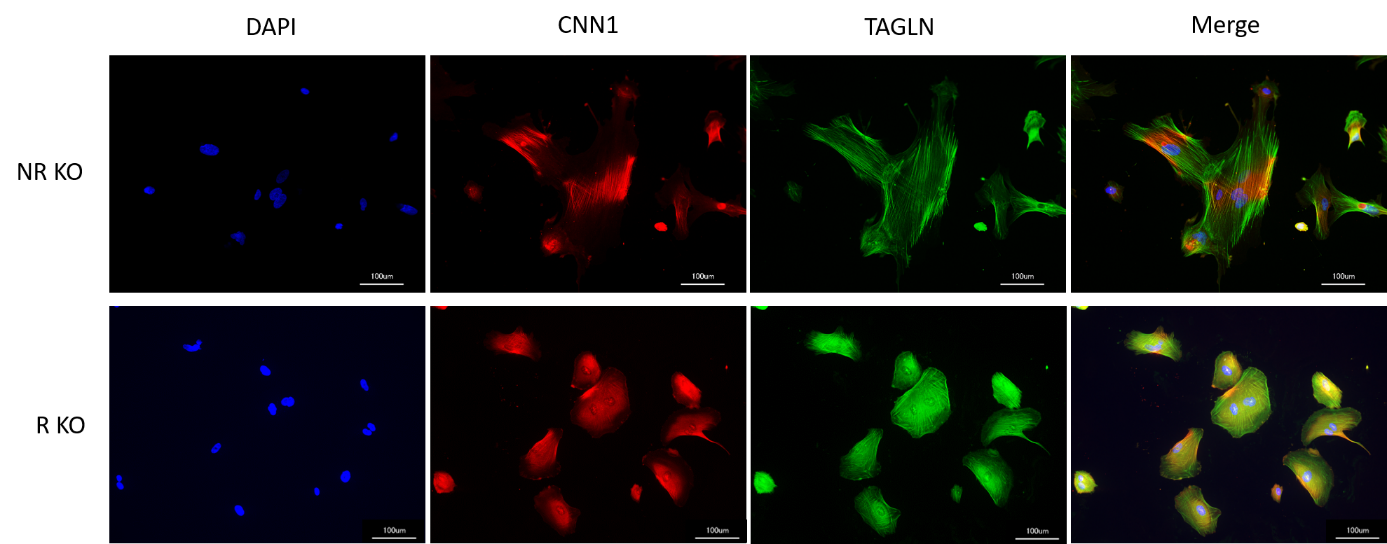


**Suppl. Fig. 2: The 9p21 locus does not influence VSMC morphology or protein localization.**

NR (Top) and R (bottom) KO iPSC-derived VSMCs show colocalization of CNN1 (red) and TAGLN (green) in the contractile apparatus of the cells. There were no differences between R and NR KO VSMCs. Nuclei were counterstained with DAPI. Scale bars = 50 µm.

Suppl. Fig. 3


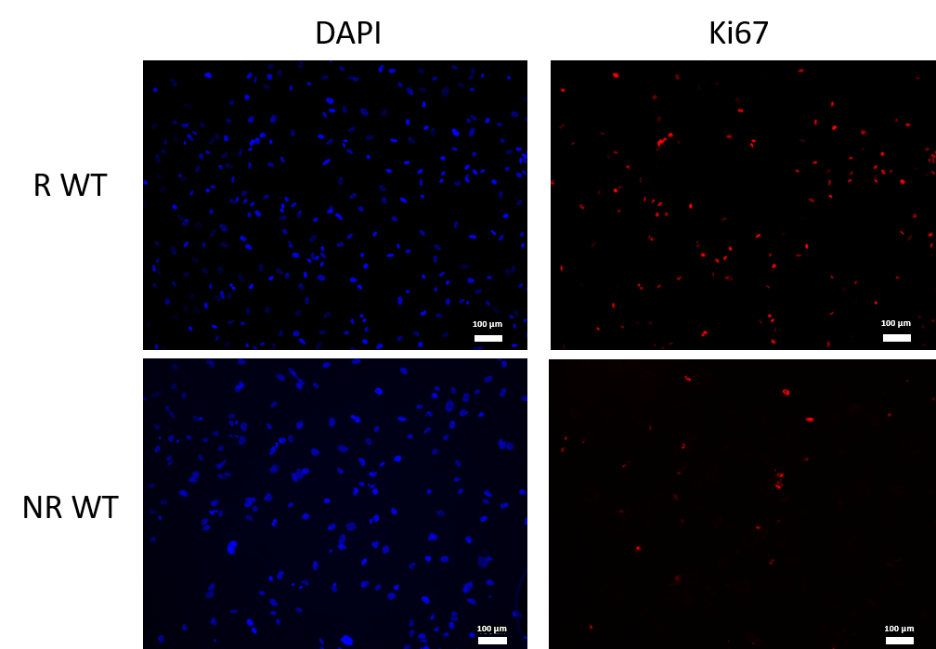


**Suppl. Fig. 3: Representative images of Ki67 staining in R vs NR WT VSMCs.**

R WT (Top) iPSC-derived VSMCs showed more Ki67+ cells compared to the NR WT (Bottom) VSMCs. Nuclei were counterstained with DAPI. Scale bars = 100 µm.

Suppl. Fig. 4


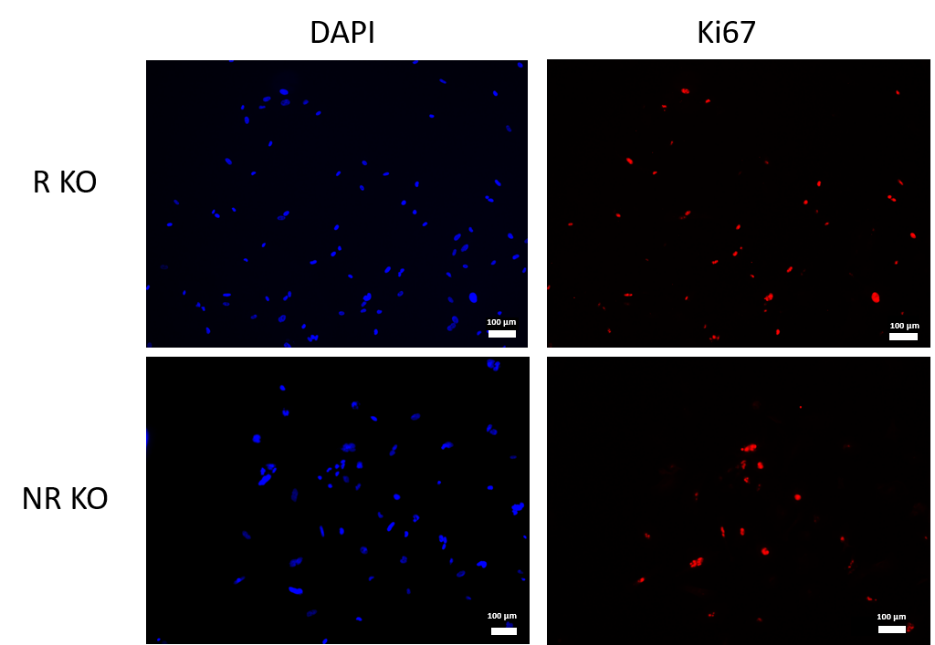


**Suppl. Fig. 4: Representative images of Ki67 staining in R vs NR KO VSMCs.**

R KO (Top) iPSC-derived VSMCs showed more Ki67+ cells compared to the NR KO (Bottom) VSMCs. Nuclei were counterstained with DAPI. Scale bars = 100 µm.

Suppl. Fig. 5


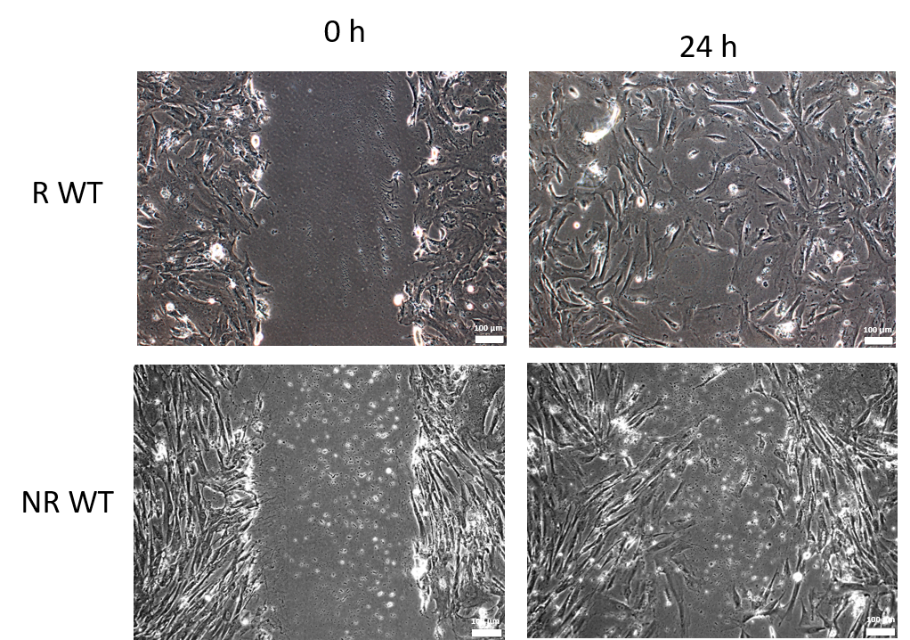


**Suppl. Fig. 5: Representative images of migration of R vs NR WT VSMCs.**

R WT (Top) iPSC-derived VSMCs showed stronger migration compared to the NR WT (Bottom) VSMCs. Scale bars = 100 µm.

Suppl. Fig. 6


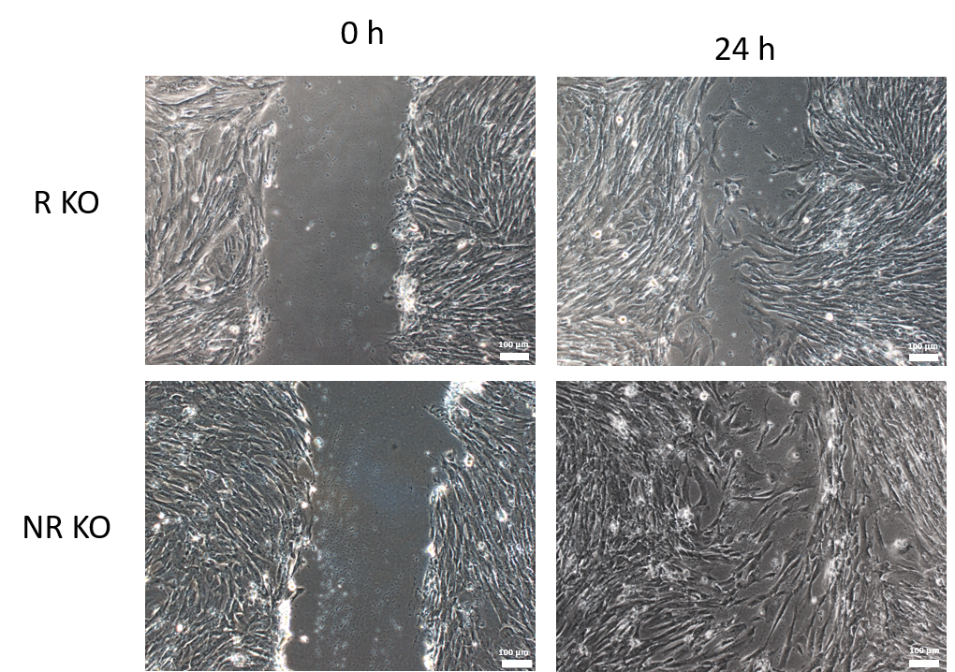


**Suppl. Fig. 6: Representative images of migration of R vs NR KO VSMCs.**

R KO (Top) and NR KO (Bottom) iPSC-derived VSMCs showed a similar migration rate. Scale bars = 100 µm.

Suppl. Fig. 7


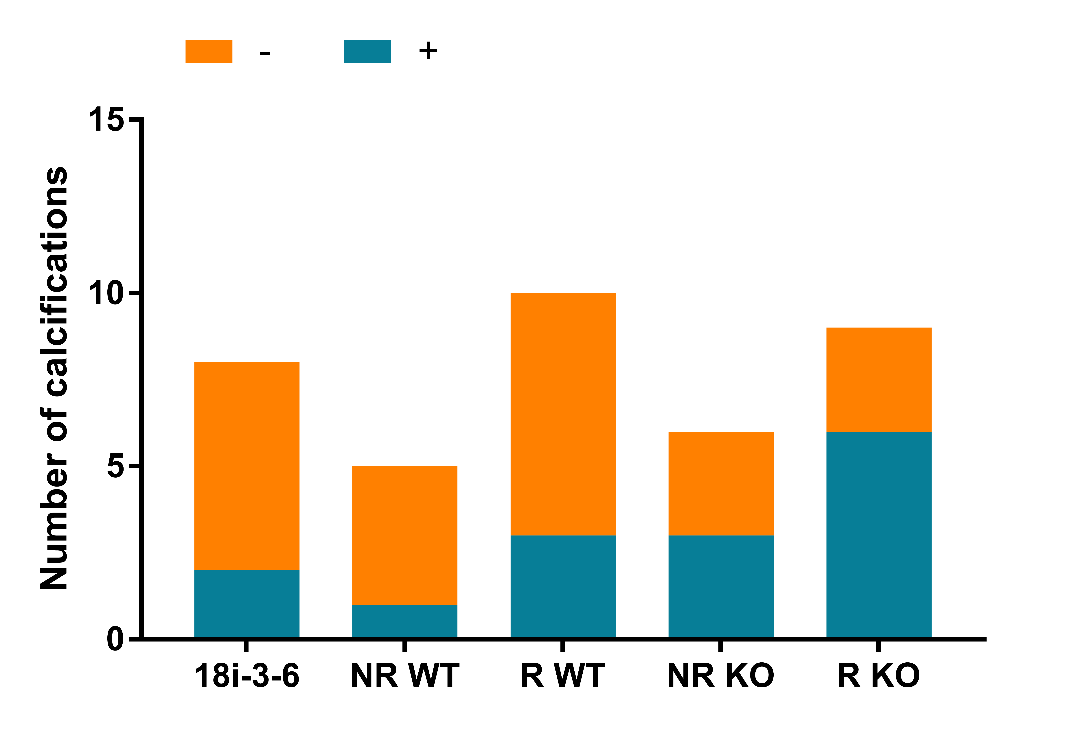


**Suppl. Fig. 7: Efficiency of Tziakas calcification cocktail over genotypes.**

The Tziakas cocktail showed the highest calcification efficiency (+; green) in the R KO and NR KO iPSC lines, but lower efficiency in R WT. The 18i-3-6 control iPSCs and NR WT showed positive calcifications.

Suppl. 1: Python Script for Calcein quantification (by Dr. Tobias Reinberger)

"""

Tobias Schöne, 23.07.2019

gets DAPI and green Fl. as TIF

give excel sheet with intensity of fluorescence and pixel count

normalized by DAPI Pixel count

To Do: delete [255 255 255] pixel

"""

import cv2

import numpy as np

import matplotlib.pyplot as plt

from openpyxl import load_workbook

import os

import datetime

today = str(datetime.date.today())

today = today.replace('-', '')

time = str(datetime.datetime.now().time()).replace(":", '_')

time = time[0:time.find('.')]

storage = "//storage.iieg.uni-luebeck.de/public/6_Keyence/Anja/Calcein/Calcein Quantifizierung/"

channels = {"CH1": ["Dapi", 0],

            "CH2": ["Calc", 1]}

threshold_dapi = 35

threshold_calein = 20

wb = load_workbook('./EMPTY.xlsx')

ws = wb.active  # sheet has to be active

ws.cell(1, 1).value = "Dateiname"

ws.cell(1, 2).value = "Intensity\n(raw DAPI)"

ws.cell(1, 3).value = "Pixel count DAPI\n(I(DAPI)/count(DAPI)"

ws.cell(1, 4).value = "norm DAPI"

ws.cell(1, 5).value = "Intensity\n(raw Calcein)"

ws.cell(1, 6).value = "Pixel count Calcein"

ws.cell(1, 7).value = "norm Calcein\n(I(Calcein)/count(DAPI))"

""" get file names """

file_array = []

for file in os.listdir(storage):

    if ".TIF" in file or ".tif" in file:

        file_array.append(file)

    pass

file_array = sorted(file_array)

print('Files:', len(file_array))

for elem in file_array:

    print(elem)

print("\n\n")

counter = 2

for file in file_array:

    if ".TIF" in file or ".tif" in file:

        image = cv2.imread(storage + file)

        plt.imshow(image)

        # plt.show()

        image_temp = np.asarray(image)

        if "CH1" in file:

            print(counter)

            print(file)

            image_temp[image_temp < threshold_dapi] = 0

            sum_ = np.sum(image_temp)

            pixel_count = np.count_nonzero(image_temp)

            ws.cell(counter, 1).value = file.replace("_CH1.TIF", "")

            ws.cell(counter, 2).value = sum_

            ws.cell(counter, 3).value = pixel_count

            ws.cell(counter, 4).value = round((sum_/pixel_count), 2)

            print("Intensity", sum_, "Pixel count", pixel_count, sum_/pixel_count)

        if "CH2" in file:

            print(file)

            image_temp[image_temp < threshold_calein] = 0

            sum_ = np.sum(image_temp)

            pixel_count_2 = np.count_nonzero(image_temp)

            ws.cell(counter, 5).value = sum_

            ws.cell(counter, 6).value = pixel_count_2

            ws.cell(counter, 7).value = round((sum_/pixel_count), 2)

            counter += 1

            print("Intensity", sum_, "Pixel count", pixel_count_2, sum_/pixel_count, "\n\n")

wb.save(storage + "%s_Calcein_Quantification.xlsx" % today)

wb.close()

Suppl. Fig. 8


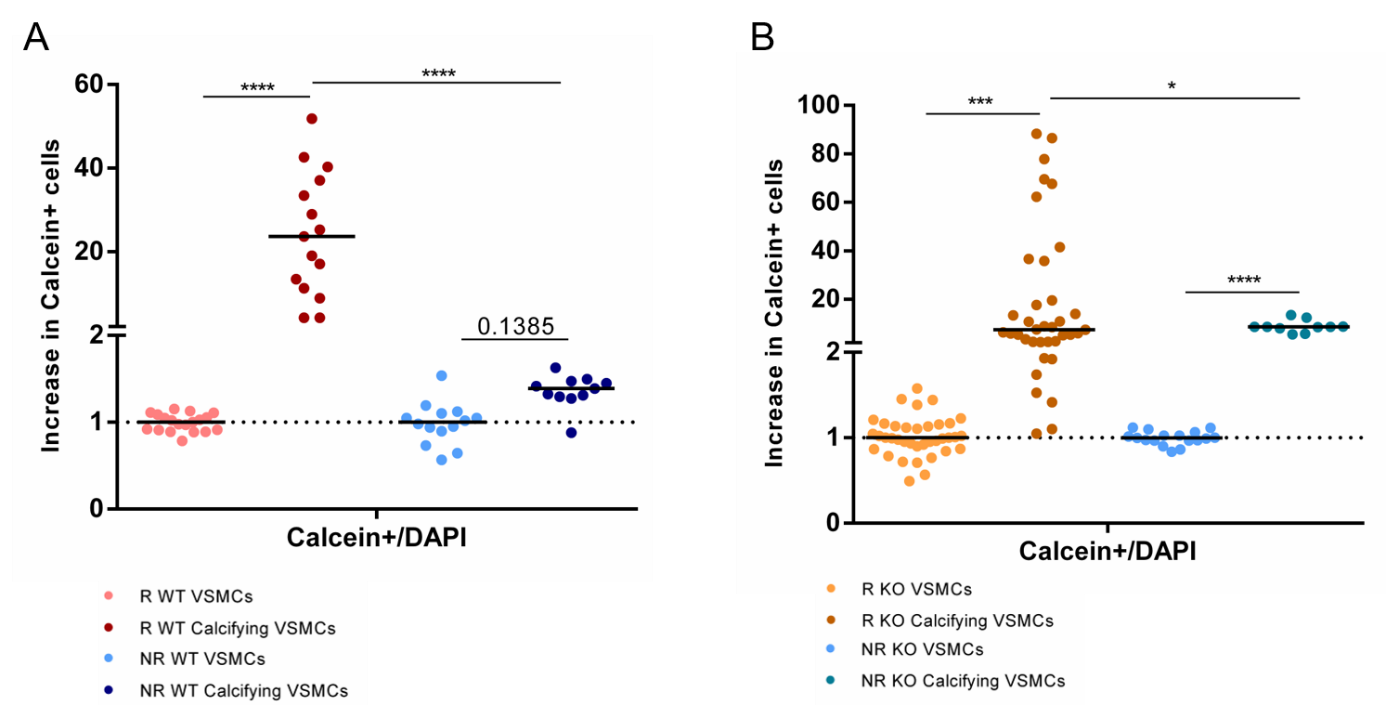


**Suppl. Fig. 8: Calcein quantifications had increased Ca levels in R WT, and both KO genotypes.**

1. R WT (red)-derived calcifying VSMCs showed significantly increased Ca levels compared with NR WT (blue) -derived cells, which showed no change.
2. R KO- (brown) and NR KO- (blue)-derived calcifying VSMCs showed significantly increased Ca levels compared with untreated VSMCs.
